# Using Anti-Malondialdehyde Modified Peptide Autoantibodies to Import Machine Learning for Predicting Coronary Artery Stenosis in Taiwanese Patients with Coronary Artery Disease

**DOI:** 10.3390/diagnostics11060961

**Published:** 2021-05-26

**Authors:** Yu-Cheng Hsu, I-Jung Tsai, Hung Hsu, Po-Wen Hsu, Ming-Hui Cheng, Ying-Li Huang, Jin-Hua Chen, Meng-Huan Lei, Ching-Yu Lin

**Affiliations:** 1 Cardiovascular Center, Lo-Hsu Medical Foundation Luodong Poh-Ai Hospital, Yilan 26546, Taiwan; 897016mmsyc@gmail.com; 2 Ph.D. Program in Medical Biotechnology, College of Medical Science and Technology, Taipei Medical University, Taipei 11031, Taiwan; d609108005@tmu.edu.tw; 3 Medical Quality Department, Lo-Hsu Medical Foundation Luodong Poh-Ai Hospital, Yilan 26546, Taiwan; endeahsu1209@gmail.com; 4 Preventive Medical Center, Lo-Hsu Medical Foundation Luodong Poh-Ai Hospital, Yilan 26546, Taiwan; sh46913.sh46913@msa.hinet.net; 5 Department of Laboratory Medicine, Lo-Hsu Medical Foundation Luodong Poh-Ai Hospital, Yilan 26546, Taiwan; 91a018@gmail.com; 6 Section of Laboratory, Lo-Hsu Medical Foundation Lodong Poh-Ai Hospital, Yilan 26546, Taiwan; daodai38@gmail.com; 7 Graduate Institute of Data Science, College of Management, Taipei Medical University, Taipei 11031, Taiwan; jh_chen@tmu.edu.tw; 8 Statistics Center, Institutional Research Center, Office of Data Science, Taipei Medical University, Taipei 11031, Taiwan; 9 School of Medical Laboratory Science and Biotechnology, College of Medical Science and Technology, Taipei Medical University, Taipei 11031, Taiwan

**Keywords:** cardiovascular disease, malondialdehyde, autoantibody isotype, plasma

## Abstract

Machine learning (ML) algorithms have been applied to predicting coronary artery disease (CAD). Our purpose was to utilize autoantibody isotypes against four different unmodified and malondialdehyde (MDA)-modified peptides among Taiwanese with CAD and healthy controls (HCs) for CAD prediction. In this study, levels of MDA, MDA-modified protein (MDA-protein) adducts, and autoantibody isotypes against unmodified peptides and MDA-modified peptides were measured with enzyme-linked immunosorbent assay (ELISA). To improve the performance of ML, we used decision tree (DT), random forest (RF), and support vector machine (SVM) coupled with five-fold cross validation and parameters optimization. Levels of plasma MDA and MDA-protein adducts were higher in CAD patients than in HCs. IgM anti-IGKC^76–99^ MDA and IgM anti-A1AT^284–298^ MDA decreased the most in patients with CAD compared to HCs. In the experimental results of CAD prediction, the decision tree classifier achieved an area under the curve (AUC) of 0.81; the random forest classifier achieved an AUC of 0.94; the support vector machine achieved an AUC of 0.65 for differentiating between CAD patients with stenosis rates of 70% and HCs. In this study, we demonstrated that autoantibody isotypes imported into machine learning algorithms can lead to accurate models for clinical use.

## 1. Introduction

Coronary artery disease (CAD) is the largest cause of death in developed countries including Taiwan [1]. In 2015, age-adjusted acute myocardial infarction (AMI) incidence which is most resulted from CAD event was 73.4 per 100,000 for men and 27.9 per 100,000 for women in Taiwan [2]. The Framingham Heart Study summarized major risk factors for CAD, which included age, inactivity, obesity, hypertension, smoking, diabetes, and gender [3]. Some risk factors may affect CAD through several mechanisms related to the formation of atherosclerosis; for instance, aging induces oxidative stress in endothelial cells as was found in rats [4]. In addition, Kianoush S. et al. observed strong associations of the smoking status and intensity of inflammation with subclinical atherosclerosis [5]. Hence, previous studies indicated that oxidative stress and inflammation levels in the vascular system may predispose patients to atherosclerosis [6,7].

Endothelial damage to the vascular system causes infiltration and accumulation of low-density lipoprotein (LDL) in the subendothelial space [8]. LDL becomes oxidized to form oxidized (Ox)-LDL [9]. Further, the accumulation of Ox-LDL can be considered as a proinflammatory factor which leads monocytes to migrate into the vascular intima and transform into macrophages [10]. Macrophages serve as the main source of foam cells, a hallmark of atherosclerosis, after they ingest and accumulate Ox-LDL [10]. In addition, Ox-LDL trapped in the intima can enhance lipid peroxidation which may increase oxidative stress and inflammation levels in the intima [11].

Many studies showed that highly reactive aldehydes derived from lipid peroxidation are strongly related to CAD events [12,13,14]. Malondialdehyde (MDA), 4-hydroxylnoneal (4-HNE), and acrolein are generated, while lipids are attacked by reactive oxygen species (ROS) [15]. Further, MDA can spread oxidative damage and form oxidation-specific epitopes (OSEs) with proteins [16]. OSEs can present on Ox-LDL, cell debris, apoptotic cells, and modified proteins in vessel walls [17]. The accumulation of OSEs can generate disease-associated antigens and strong proinflammatory molecules [16]. Moreover, OSEs play important roles in physiological processes and are able to serve as markers of oxidative-modified structures, which allows the immune system to regulate their clearance and mediate homeostasis [17,18]. In fact, both the cellular and humoral immune systems are involved in the progression and development of CAD through atherosclerosis [19]. For example, Bartolini G. et al. reported that immunoglobulin M (IgM) was shown to block MDA-epitopes and other OSEs from macrophages to reduce generation of foam cells in atherosclerosis [20]. In addition, increasing levels of IgG against unmodified proteins such as high-density lipoprotein (HDL) and serum paraoxonase and arylesterase 1 (PON1) were previously reported in patents with CAD [21]. Moreover, to evaluate atherosclerosis-related disease, circulating autoantibodies against atherosclerosis-related antigens were considered to be novel biomarkers [22].

CAD is the second leading cause of death of patients with rheumatoid arthritis (RA) in Taiwan [23]. Further, CAD progression is strongly related to the immune response [24]. Moreover, in the previous study carried out by Liao C.C. et al. suggested that IgG and IgM isotypes against four different novel MDA-modified peptides (Ig kappa chain C region (IGKC^76–99^), alpha-1-antitrypsin (A1AT^284–298^), alpha-2-macroglobulin (A2M^824–841^), and apolipoprotein B-100 (ApoB100^4022–4040^) can differentiate patients with RA and healthy controls (HCs) with an AUC of 0.96–0.98 [25]. Therefore, we speculated that the autoantibody isotypes against four different novel MDA-modified peptides identified in patients with RA may be related to CAD events. In this study, we first examined levels of IgG and IgM isotypes against MDA-modified bovine serum albumin (BSA) in HCs, in patients with RA, and in patients with RA and CAD. Next, we detected MDA levels and MDA protein adducts (MDA proteins) in HCs and patients with CAD to evaluate their oxidative stress levels. Further, we determined levels of IgG and IgM antibodies against four different unmodified peptides and MDA-modified peptides in HCs and patients with CAD (<30%, 30–70%, >70%). Lastly, we chose three different algorithms, including a decision tree (DT), random forest (RF), and support vector machine (SVM) in Scikit-Learn (Vers. 0.21.3) to build models for predicting the stenosis rate with five-fold cross validation. Forward selection was performed in this study to select the most optimal autoantibody combinations. The sensitivity, specificity, and area under the receiver operating characteristics (ROC) curve (AUROC) were used to evaluate the performance of the models.

## 2. Materials and Methods

### 2.1. Patient Samples

Serum samples from 30 patients with RA (18 female and 12 male patients, aged 56.43 ± 8.29 years) and 30 patients with RA and diagnosed as CAD (21 female and nine male patients, aged 56.26 ± 8.29 years) were obtained from the Division of Allergy, Immunology, and Rheumatology, Department of Internal Medicine and the Department of Laboratory Medicine, Shuang-Ho Hospital (NTPC, Taiwan). Patients with RA and those with RA and CAD received diagnoses from a rheumatologist following criteria of either the 2010 American College of Rheumatology (ACR)/European League Against Rheumatism classification criteria or 1987 ACR classification criteria. Patients with RA and CAD were defined as those patients with RA who visited the Division of Cardiology department. Plasma samples from 172 patients with CAD (48 female and 124 male patients, aged 62.99 ± 9.59 years) and 40 HCs (16 females and 24 males, aged 38.41 ± 10.42 years) were obtained from the Cardiovascular Center and Department of Laboratory Medicine, Lo-Hsu Medical Foundation Luodong Poh-Ai Hospital (ILH, Taiwan). Patients with CAD were diagnosed by a cardiologist via a coronary angiogram test. Acute coronary syndrome (ACS) was not included in this study. Patients with CAD were divided into three classes by the coronary artery stenosis rate: <30%, 30%~70%, and >70%. This study was approved by the institutional review board of the study hospital, and all volunteers provided informed consent before participating. Patient samples were randomly selected and age-paired with HCs. Clinical and demographic characteristics of CADs and HCs are presented in Table 1. Plasma samples were stored at −80 °C until being analyzed. Lipid profiles are important in the development of CAD. Thus, we further analyzed triglycerides (TGs), LDL, high-density lipoprotein (HDL), and total cholesterol (CHOL) with a point of care testing (POCT) machine from Skyla (HCT, Taiwan) following manufacturers’ instructions. The Institutional Review of Cathay General Hospital and the Taipei Medical University-Joint Institutional Review Board approved the study protocol, and all volunteers signed an informed consent form before participating in the study (CGP-LP106006 (15 June 2017), N201512049 (3 February 2017)). The study conforms to the ethical guidelines of the 1975 Declaration of Helsinki.

### 2.2. Detection of Plasma MDA and MDA Protein Adducts

To detect MDA levels, we conducted a thiobarbituric acid-reactive substance (TBARS) assay. The experiment followed Costa’s protocol [26]. To quantify MDA protein adducts, we followed the protocol of Schutt et al. [27]. All experiments on samples were duplicated. Details are provided in “Supplementary Information”.

### 2.3. Detection of Plasma Autoantibodies against Unmodified and Modified Peptides

Four different MDA-modified peptides were identified in our previous study. In brief, serum from patients with RA was purified with agarose-bound concanavalin (Con) A chromatography. Purified serum samples were analyzed with nano liquid chromatography-tandem mass spectroscopy (nanoLC-MS/MS). Four MDA-modified peptides were identified with PEAKS in-house. Four different MDA-modified peptides were discovered, including Ig kappa chain C region (IGKC^76–99^, ADYEK**HK**VYACEVTHQGLSSPVTK), alpha-1-antitrypsin (A1AT^284–298^, LQHLENELT**H**DIITK), alpha-2-macroglobulin (A2M^824–841^, VSVQLEASPAFLAVPVE**K**), and apolipoprotein B-100 (ApoB100^4022–4040^, WNFYYSPQSSPD**KK**LTIF**K**). Further, immunoprecipitation (IP)-Western blotting was conducted to examine modifications of the MDA proteins. In general, IgG and IgM isotypes against unmodified and MDA-modified IGKC^76–99^, A1AT^284–298^, A2M^824–841^, and ApoB100^4022–4040^ were significantly higher in serum derived from patients with RA and OA.

To detect IgG and IgM isotypes against unmodified and MDA-modified peptides, including IGKC^76–99^, A1AT^284–298^, A2M^824–841^, and ApoB100^4022–4040^, 213 plasma samples were examined by an enzyme-linked immunosorbent assay (ELISA) [25]. Polypeptides were synthesized (Yao-Hong Biotechnology, NTPC, Taiwan) and used for the ELISA. MDA-modified peptides were prepared with malonaldehyde bis (S32088 843, Millipore, MA, USA) [24]. All experiments on samples were run in duplicate. Details are provided in “Supplementary Materials”.

### 2.4. Statistical Analysis

The significance of MDA protein adducts and levels of autoantibody isotypes against unmodified and MDA-modified peptides between HCs and patients with CAD were determined with Student’s *t*-test and a one-way analysis of variance (ANOVA). Student’s *t*-test was calculated with GraphPad Prism (v.5.0; GraphPad Software, SD, CA, USA). Logistic regression models were used to estimate multivariate-adjusted odds ratios (ORs) and their 95% confidence intervals (CIs) for patients with a >30% stenosis rate risk of CAD. The logistic regression models were used to estimate the association between MDA levels, MDA adducts, and autoantibodies isotype between subjects with <30% stenosis rate and subjects >30% stenosis rate. The cut-off values were set as 25th percentile in variables. The one-way ANOVA and ROC curve analysis were calculated using SAS (v.9.3; SAS Institute, Cary, NC, USA). The significance level of all statistical tests was set to *p* < 0.05. Here, we evaluated our model based on decision trees classifier (DT), random forests classifier (RF), and support vector machine (SVM) with five-fold cross validation in Scikit-Learn (v.0.21.3). Parameter tuning was performed for each training and validation set on the basis of the five-fold cross validation. The tunning process was based on value of AUC. For DT, we used initial value of tree depth was set to 1–10 with a step of 1. The kernel of model was set to Gini or entropy. As for RF, the initial value of tree number was set to 100 and increased by 100 until 500. The kernel of model was set to Gini or entropy. For SVM, the initial value of gamma was set to 10^−6^–10^−10^ with a step of 10^−1^. The initial value of C was set to 10^6^–10^7^ with a step of 10-fold. The kernel of model was set to RBF. Lastly, we applied a confusion matrix to calculate the accuracy, sensitivity, specificity, and AUC.

## 3. Results

### 3.1. Determination of Autoantibodies against MDA-Modified BSA in RA with CAD Patients

Four different MDA-modified peptides were discovered from RA patients in our previous study [25]. In this study, plasma samples were subjected to an ELISA to determine isotypes of autoantibodies against unmodified and MDA-modified BSA. Plasma levels of IgG and IgM against BSA were found not to differ among patients with CAD, those with RA and CAD, and HCs (Supplementary Figure S1A,B, left panel). In contrast, plasma levels of IgM against MDA-modified BSA were higher in RA with CAD patients and RA patients compared with HCs (Supplementary Figure S1B, right panel). Thus, to explore the potential for IgG and IgM isotypes against MDA-modified peptides to serve as biomarkers in CAD, we further validated MDA, MDA protein adducts, and antibody isotypes in plasma derived from patients with CAD.

### 3.2. Detection of MDA and MDA Protein Adducts

In order to determine oxidative stress in plasma samples, we analyzed TBARSs to detect MDA levels. Plasma levels of MDA in patients with CAD with >70% stenosis were higher than those of patients with CAD with <30% stenosis (1.12-fold), as well as those with CAD with 30%~70% stenosis (1.01-fold), and HCs (1.26-fold, Table 1). Furthermore, plasma samples were subjected to an ELISA to determine MDA protein adducts in plasma samples. Plasma levels of MDA protein adducts in patients with CAD were higher than those of HCs (Table 1).

### 3.3. Measuring of Autoantibodies against Unmodified and MDA-Modified Peptides

Plasma samples were assessed through an ELISA using isotypes of autoantibodies against unmodified and MDA-modified peptides (Supplementary Figure S2). We further used an ANOVA and Student’s *t*-test to examine the significance among patients with CAD with <30% stenosis, those with CAD with 30~70% stenosis, those with CAD with >70% stenosis, and HCs. Scheffe’s post-hoc test was used to determine the significance between each two groups.

Plasma levels of IgG against the A2M^824–841^ unmodified and MDA-modified peptides among patients with CAD and HCs did not significantly differ (Supplementary Figure S2A). Plasma levels of IgM against the A2M^824–841^ unmodified peptide among patients with CAD with >70% stenosis were lower than those of HCs by 0.58-fold (*p* < 0.0001), patients with CAD with 30~70% stenosis were 0.66-fold (*p* < 0.0001) lower than HCs, and patients with CAD with <30% stenosis were 0.69-fold (*p* = 0.0027) lower than HCs (Supplementary Figure S2E). Plasma levels of IgM against the A2M^824–841^ MDA-modified peptides among patients with CAD with >70% stenosis were lower than those of HCs by 0.54-fold (*p* < 0.0001), patients with CAD with 30~70% stenosis were 0.69-fold (*p* = 0.002) lower than HCs, and patients with CAD with <30% stenosis were 0.65-fold (*p* = 0.001) lower than HCs (Supplementary Figure S2E, right panel). The ROC curve analysis of autoantibodies against A2M^824–841^ unmodified peptide and A2M^824–841^ MDA-modified peptide are summarized in Supplementary Figure S3 and Supplementary Table S2.

Plasma levels of IgG against the ApoB100^4022–4040^ unmodified peptide between patients with CAD with >70% stenosis and HCs were, significantly, 0.72-fold (*p* = 0.0075) lower (Supplementary Figure S2B, left panel). However, levels of IgG against the ApoB100^4022–4040^ MDA-modified peptide did not differ among patients with CAD and HCs (Supplementary Figure S2B, right panel). Levels of IgM against the ApoB100^4022–4040^ unmodified peptide did not differ between patients with CAD and HCs (Supplementary Figure S2F, left panel). In contrast, IgM levels against the ApoB100^4022–4040^ MDA-modified peptide between patients with CAD with >70% stenosis and HCs were significantly 0.60-fold (*p* = 0.0003) lower and patients with CAD with >70% stenosis versus patients with CAD with <30% stenosis were 0.79-fold (*p* = 0.0388) lower (Supplementary Figure S2F, right panel). The ROC curve analysis of autoantibodies against ApoB100^4022–4040^ unmodified peptide and ApoB100^4022–4040^ MDA-modified peptide were summarized in Supplementary Figure S3 and Supplementary Table S2.

Plasma levels of IgG against A1AT^284–298^ unmodified and MDA-modified peptides did not significantly differ between patients with CAD and HCs (Supplementary Figure S2C). Plasma levels of IgM against the A1AT^284–298^ unmodified peptide between patients with CAD with >70% stenosis were 0.53-fold (*p* = 0.0047) lower than those of HCs (Supplementary Figure S2G). Further, plasma levels of IgM against the A1AT^284–298^ MDA-modified peptide among patients with CAD with 70% stenosis were lower than HCs by 0.48-fold (*p* < 0.0001), CAD with 30~70% stenosis were lower than HCs by 0.68-fold (*p* = 0.0003), and patients with CAD with <30% stenosis were lower than HCs by 0.71-fold (*p* = 0.0025) (Supplementary Figure S2G, right panel). The ROC curve analysis of autoantibodies against A1AT^284–298^ unmodified peptide and A1AT^284–298^ MDA-modified peptide are summarized in Supplementary Figure S3 and Supplementary Table S2.

Plasma levels of IgG against the IGKC^76–99^ unmodified peptide between patients with CAD with >70% stenosis were 0.66-fold (*p* = 0.0356) lower than those of HCs, and patients with CAD with >70% stenosis were 0.60-fold (*p* = 0.0413) lower than those of patients with CAD with <30% stenosis (Supplementary Figure S2D, left panel). Plasma levels of IgG against the IGKC^76–99^ MDA-modified peptide between patients with CAD with >70% stenosis were significantly lower than those of HCs (Supplementary Figure S2D right panel). Plasma levels of IgM against the IGKC^76–99^ unmodified peptide between patients with CAD with >70% stenosis were significantly 0.52-fold (*p* = 0.0009) lower than those of HCs, and patients with CAD with 30~70% stenosis were 0.58-fold (*p* = 0.0122) lower than those of HCs (Supplementary Figure S2H, left panel). Plasma levels of IgM against the IGKC^76–99^ unmodified peptide of patients with CAD with >70% stenosis were 0.60-fold (*p* = 0.017) lower than those of HCs. Plasma levels of IgM against the IGKC^76–99^ MDA-modified peptide of patients with CAD with >70% stenosis were significantly 0.48-fold (*p* < 0.0001) lower than those of HCs, patients with CAD with 30~70% stenosis were 0.63-fold (*p* = 0.0001) lower than those of HCs, and patients with CAD with <30% stenosis were 0.62-fold (*p* = 0.0083) lower than those of HCs (Supplementary Figure S2H, right panel). The ROC curve analysis of autoantibodies against IGKC^76–99^ unmodified peptide and IGKC^76–99^ MDA-modified peptide are summarized in Supplementary Figure S3 and Supplementary Table S2.

### 3.4. Associations of Plasma Autoantibodies against Unmodified and MDA-Modified Peptides

We performed a logistic regression model adjusted for age, sex, and smoke to calculate ORs of MDA levels, MDA adduct levels, and autoantibody levels in CAD development. In the calculation of ORs, we defined HCs and patients with CAD with <30% stenosis rate as a relatively lower risk group while patients with CAD with 30~70% and >70% stenosis rate as a relatively higher risk group. The baseline characteristic of two groups were provided in Supplementary Table S1. As shown in Supplementary Table S2, ORs of CAD development were significantly associated with autoantibodies to unmodified and MDA-modified peptides in patients with a >30% stenosis rate compared to individuals with a stenosis rate of <30%: MDA (OR = 2.149, *p* = 0.046), MDA adduct (OR = 0.562, *p* = 0.129), IgG anti A2M^824–841^ (OR = 2.022, *p* = 0.05), IgG anti A2M^824–841^ MDA (OR = 1.076, *p* = 0.842), IgG anti-ApoB100^4022–4040^ (OR = 0.315, *p* = 0.004), IgG anti-ApoB100^4022–4040^ MDA (OR = 0.705, *p* = 0.360), IgG anti-A1AT^284–298^ (OR = 1.446, *p* = 0.304), IgG anti-A1AT^284–298^ MDA (OR = 1.739, *p* = 0.127), IgG anti-IGKC^76–99^ (OR = 0.578, *p* = 149), IgG anti-IGKC^76–99^ MDA (OR = 0.663, *p* = 266), IgM anti-A2M^824–841^ (OR = 0.311, *p* = 0.004), IgM anti-A2M^824–841^ MDA (OR = 0.533, *p* = 0.105), IgM anti-ApoB100^4022–4040^ (OR = 0.580, *p* = 0.157), IgM anti-ApoB100^4022–4040^ MDA (OR = 0.288, *p* = 0.002), IgM anti-A1AT^284–298^ (OR = 0.356, *p* = 0.010), IgM anti-A1AT^284–298^ MDA(OR = 0.191, *p* < 0.001), IgM anti-IGKC^76–99^ (OR = 0.905, *p* = 0.790), and IgM anti-IGKC^76–99^ MDA (OR = 0.485, *p* = 0.072). The IgM anti-A1AT^284–298^ MDA was the strongest protective factor of CAD (Table 2). In contrast, IgG anti A2M^824–841^ carried the highest risk of CAD (Table 2). The OR results were only considered significant if the *p* value was <0.05, including IgG anti-ApoB100^4022–4040^, IgM anti-A2M^824–841^, IgM anti-ApoB100^4022–4040^ MDA, IgM anti-A1AT^284–298^ MDA, IgM anti-A1AT^284–298^.

### 3.5. Using Plasma Anti-Unmodified and Anti-MDA-Modified Peptide Autoantibodies to Identify CAD Patients from HCs

**Figure 1:**
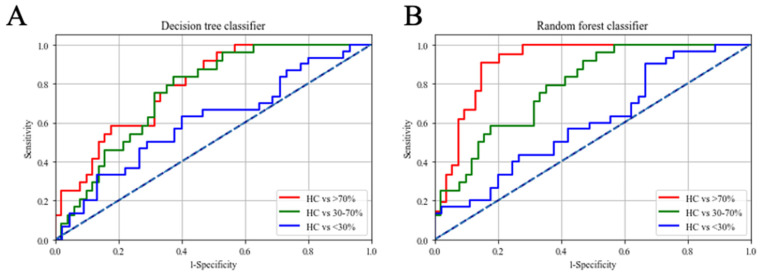
Comparison of the area under the receiver operating characteristic curve (AUC) of autoantibody isotypes against unmodified and malondialdehyde (MDA)-modified peptides in coronary artery disease (CAD) patients compared to healthy controls (HCs) with a decision tree classifier (**A**) and random forest classifier (**B**).

To further explore the potential of our autoantibodies examined in plasma to identify patients with CAD, we built three models with a DT, RF, and SVM. Predictive performances based on forward selected autoantibodies are summarized in Figure 1 and Table 3. We found 0.67, 0.76, and 0.81 values for the area under the curve (AUC) (HC vs. <30%, HC vs. 30~70%, and HC vs. >70%, respectively) with the DT as shown in Figure 1A and Table 3. Further, IgM anti-ApoB^4022–4040^ MDA, IgM anti-IGKC^76–99^ MDA, and IgM anti-A1AT^284–298^ MDA were selected as the best attributes to classify patients with CAD from HCs. As to the RF, we found 0.76, 0.91, and 0.94 values of the AUC (HC vs. <30%, HC vs. 30%~70%, and HC vs. >70%, respectively) as shown in Figure 1B and Table 3. Moreover, IgG anti-IGKC^76–99^, IgM anti-IGKC^76–99^ MDA, and IgM anti-A1AT^284–298^ MDA were selected as the best attributes to classify patients with CAD from HCs. However, we found poor results with the SVM; we found 0.55, 0.56, and 0.65 values of the AUC (HC vs. <30%, HC vs. 30~70%, and HC vs. >70%, respectively) with the SVM (data not shown). Our results showed that the RF can distinguish CAD patients with 30%~70% and >70% stenosis from HCs with fewer attributes and better performance.

## 4. Discussion

The major findings of this study are that levels of autoantibodies of IgG and IgM isotypes against MDA-modified peptide adducts were significantly lower in patients with CAD. Further, strong associations were observed of the auto-antibodies IgM anti-IGKC^76–99^ MDA and IgM anti-A1AT^284–298^ MDA with CAD. Previous studies examined the association of CAD and seropositive patients; for instance, a 3.6-fold increased risk of death from CAD was reported in a cohort study of RA patients [28]. Further, excessive autoantibodies produced by patients with RA were associated with high inflammation, which contributed to endothelial dysfunction, plaque vulnerability, and accelerated atherosclerosis [29]. Therefore, we speculated the autoantibodies against MDA modified peptide discovered in patients with RA in a previous study may be useful in diagnosis CAD. In our study, autoantibody isotypes of IgM and IgG against MDA-modified BSA in RA patients with CAD were higher than in RA patients and HCs (Supplementary Figure S1). Differences among groups indicated that autoantibody isotypes against MDA modified peptide are related to CAD progression.

MDA is one of the toxic byproducts of lipid peroxidation [30]. In addition to lipid peroxidation, enzymatic processes from prostaglandins can also generate MDA in vivo [31]. Excessive MDA in the blood can damage tissues and increase the risk of CAD; for example, intermolecular cross-linking of collagen via MDA may significantly stiffen vascular tissues [30]. In addition, a correlation between MDA and CAD was observed in an elegant study carried out by Hadj Adhmed S. et al. [32]. In our study, MDA and MDA protein adduct levels were higher in patients with CAD compared with HCs (Table 1) which is consistent with previous reports [33,34].

The accumulation of MDA protein adducts can be neutralized by an individual’s immune system [18]. Miller et al. suggested that OSEs including MDA can be considered a novel class of DAMPs [35]. OSEs are recognized by PRRs such as scavenger receptors (SRs) present on macrophages, which can bind and internalize oxidized but not native LDL [36]. This consequently leads to the formation of lipid-laden foam cells [36].

Natural antibodies (NAAs) and autoantibodies refer to antibodies that bind to self-antigens, including DNA, and phospholipids [37,38]. Most natural antibodies are IgM that are poly-reactive to multiple antigens with low-affinity binding, while most autoantibodies are IgG and react to single antigens with high-affinity binding [38,39].

IgM can block the binding, uptake, and degradation of Ox-LDL by macrophages [40]. Further, IgM against OSEs can protect individuals by neutralizing the proinflammatory effects of OSEs and promote the anti-inflammatory clearance of cellular debris [41]. Moreover, many studies indicated that declining levels of IgM against MDA-modified proteins are associated with increasing risks of CAD; for example, Björkbacka H. et al. observed that subjects who suffered an acute coronary event had lower levels of IgM-p45 MDA (hazard ratio: 0.72) and lower levels of IgG-p210 native (hazard ratio: 0.73) [42]. Further, Su J. et al. suggested that individuals with high levels of IgM isotypes against phosphorylcholine (PC) (OR: 0.46), Ox-LDL (OR: 0.46), and MDA-modified LDL (OR: 0.27) can predict a decreased rate of development of carotid intima medium thickness (IMT) in patients with hypertension [43]. Moreover, Thiagarajan D. et al. discovered that an IgM isotype against MDA-modified human albumin was associated with a decreased CAD risk (OR: 0.68) and suggested that it could be a protective marker for CAD [44]. In this study, we examined whether autoantibody isotypes against MDA-modified peptides can be considered protective factors for CAD. Levels of IgM against unmodified and MDA-modified peptides were lower in CAD patients compared with HCs (Supplementary Figure S1). This finding is consistent with those reported previously [42,43,44]. Levels of IgG against unmodified and MDA-modified peptides were mostly lower in CAD patients compared with HCs (Supplementary Figure S1). However, the level of IgG anti-A2M^824–841^ was higher in CAD patients compared with HCs (Supplementary Figure S1A). A previous study reported that IgG anti-HDL and IgG anti-PON1 were higher in patients with CAD; further, the antiatherogenic effects of HDL and PON1 can be inhibited via autoantibodies [21]. Therefore, we speculated that the anti-inflammatory effect of A2M may be inhibited via IgG anti-A2M^824–841^. Palma, J. et al. suggested that the natural antibodies level may decrease while age increased, and disease occurred [45]. Therefore, we performed age, sex, and smoke-adjusted logistic regression model to assess the association between subjects with <30% stenosis rate and subjects with >30% stenosis rate. According to results of the logistic regression, IgM anti-IGKC^76–99^ MDA (OR = 0.485, *p* = 0.072), IgM anti-A2M^824–841^ (OR = 0.311, *p* = 0.004), IgG anti-ApoB100^4022–4040^ (OR = 0.315, *p* = 0.004), IgM anti-ApoB100^4022–4040^ MDA (OR = 0.288, *p* = 0.002), IgM anti-A1AT^284–298^ (OR = 0.356, *p* = 0.010), and IgM anti-A1AT^284–298^ MDA (OR = 0.191, *p* < 0.001) were found to be strongly associated with the development of CAD. Altogether, we inferred that IgM against MDA-modified peptides might be considered a protective factor in CAD development.

Machine learning methods have been increasingly used in clinical research due to their high accuracy [46]. In recent years, machine learning has been widely used as a diagnostic tool in clinical studies; Ambale-Venkatesh B. et al. conducted a large cohort study across species with a dataset combined with age, sex, weight, body fat, drug history, family history, and laboratory findings; the model they built was based on an artificial neural network and achieved 79% accuracy [47]. Further, Yang L. et al. used various models including a regression tree, naïve Bayes, Ada Boost, and RF to perform CAD predictions; the datasets they used included gender, smoking, weight, cholesterol, and glucose and achieved an AUC of 0.78 with the RF [48]. To create an accurate prediction model with machine learning algorithm, it requires large sample size for training and tunning. However, an elegant study from Vabalas A. et al. indicated that training machine learning algorithms with proper ratio of features to sample size (feature/samples < 1/3, sample size >80) can reduce the bias resulted from sample number [49]. Thus, the smallest sample size in this study was 86 (HC vs. <30%) with three features which satisfy the suggestion. In this study, three models including DT, RF, and SVM were built to predict the risk of experiencing CAD. We received good classification results (sensitivities of 74.6%, 90.2%, and 88.7%; specificities of 64.5%, 82.7%, and 85.8%; and accuracies 63.5%, 83.8%, and 87.9% for HCs vs. <30%, HCs vs. 30~70%, and HCs vs. >70% stenosis rates of CAD patients, respectively) with the RF (Figure 1B). Thus, it can be used as a diagnostic tool to detect early to mid-stages of CAD. Further, the random forest classifier has been applied to discover the association between disease and the features [50]. Moreover, in a previous study, feature selection was used to optimize the model performance [51]. In this study, IgG anti-IGKC^76–99^, IgM anti-IGKC^76–99^ MDA and IgM anti-A1AT^284–298^ MDA were selected as the most frequent during random forest classifier training with forward selection. This indicated that the selected autoantibodies were strongly associated with CAD. Altogether, the results from random forest classifier and logistic regression suggested that IgM anti-IGKC^76–99^ MDA and IgM anti-A1AT^284–298^ MDA may be involved in the development of CAD. We then compared the random forest classifier results with the ROC curve analysis of single autoantibody (Supplementary Figure S3 and Supplementary Table S2). We found that the combination of IgG anti-IGKC^76–99^, IgM anti-IGKC^76–99^ MDA and IgM anti-A1AT^284–298^ MDA had better performance in discriminating HCs and >70% stenosis rates of CAD patients compared to single autoantibody.

## 5. Conclusions

In this study, we observed that various IgG and IgM isotypes against unmodified and MDA-modified peptide adducts may be associated with the development of CAD. The random forest classifier exhibited the best performance of differentiating patients with CAD and HCs. We concluded that decreased levels of IgM anti-IGKC^76–99^ MDA and IgM anti-A1AT^284–298^ MDA were related to the development of CAD from the statistical and machine learning results.

## Figures and Tables

**Table 1:** Demographic and clinical characteristics of individual subjects contributing to plasma for healthy controls (HCs) and coronary artery disease (CAD) patients with <30%, 30~70%, and >70% stenosis rates.

Variables	Shuang-Ho Hospital		Luodong Poh-Ai Hospital			
				Stenosis Rate of Patients		
	RA (*n* = 30)	RA with CAD (*n* = 30)	HC (*n* = 40)	<30% (*n* = 46)	30–70% (*n* = 47)	>70% (*n* = 79)
Age (yr)	56.43 ± 8.29	56.26 ± 8.29	38.41 ± 10.42	62.72 ± 10.32 **	63.57 ± 9.55 **	62.79 ± 9.27 **
Male	12 (40%)	9 (30%)	24 (60%)	31 (67%)	33 (70%)	60 (75%)
Drinker	-	-	9 (22%)	7 (15%)	7 (14%)	9 (11%)
Used to smoke	-	-	0	16 (34%) *	8 (17%)	19 (24%)
Current smoker	-	-	13 (32%)	2 (4%) *	10 (21%)	28 (35%)
Diabetes	-	-	-	13 (28%)	17 (36%)	31 (39%)
Hypertension	-	-	-	28 (60%)	40 (85%)	51 (64%)
Use of lipid-lowering agents	-	-	-	14(30%)	18 (38%)	44 (55%)
TC (mg/dL)	-	-	160.03 ± 35.22	144.52 ± 37.35	142.92 ± 31.35 *	140.67 ± 45.49 *
HDL-c (mg/dL)	-	-	50.83 ± 15.41	45.83 ± 15.74	46.46 ± 16.07	39.16 ± 12.41 **
LDL-c (mg/dL)	-	-	94.09 ± 35.92	84.29 ± 30.81	85.46 ± 33.19	89.28 ± 38.42
TG (mg/dL)	-	-	77.26 ± 28.46	117.06 ± 116.06	109.25 ± 113.86	123.94 ± 104.88 *
MDA (μM)	-	-	10.1 ± 4.7	11.37 ± 3.75	12.63 ± 5.49	12.81 ± 7.64
MDA-protein adducts (μg/mL)	-	-	0.208 ± 0.016	0.219 ± 0.023 *	0.215 ± 0.021	0.216 ± 0.021 *

*p*-values by *t*-test for continuous variables and Chi2 test for categorical variables. * *p*-value < 0.05, ** *p*-value < 0.0001.

**Table 2:** Association of malondialdehyde (MDA) protein adducts and autoantibody isotypes against unmodified and MDA-modified peptides in coronary artery disease patients with a stenosis rate of >30% compared to patients with a stenosis rate of <30%.

Variables	Cut Off	Stenosis Rate		Multivariate Logistic Regression Model ^$^	
		<30%	>30%		
		*n* = 86	*n* = 126	ORs (95% C.I.)	*p*-Value
MDA	8.453	29	25	Ref.	0.046
	8.453	57	101	2.149 (1.012, 4.561)	
MDA adduct	0.202	18	38	Ref.	0.129
	0.202	68	88	0.562 (0.267, 1.183)	
IgG anti A2M^824–841^	0.706	26	27	Ref.	0.054
	0.706	60	99	2.022 (0.986, 4.15)	
IgG anti A2M^824–841^ MDA	3.118	21	32	Ref.	0.842
	3.118	65	94	1.076 (0.522, 2.219)	
IgG anti ApoB100^4022–4040^	0.990	13	40	Ref.	0.004
	0.990	73	86	0.315 (0.142, 0.701)	
IgG anti ApoB100^4022–4040^ MDA	0.582	18	35	Ref.	0.360
	0.582	68	91	0.705 (0.333, 1.492)	
IgG anti A1AT^284–298^	1.260	23	29	Ref.	0.304
	1.260	63	97	1.446 (0.716, 2.922)	
IgG anti A1AT^284–298^ MDA	2.033	25	28	Ref.	0.127
	2.033	61	98	1.739 (0.854, 3.539)	
IgG anti IGKC^76–99^	0.766	17	36	Ref.	0.149
	0.766	69	90	0.578 (0.274, 1.217)	
IgG anti IGKC^76–99^ MDA	0.677	14	38	Ref.	0.266
	0.677	72	88	0.663 (0.321, 1.37)	
IgM anti A2M^824–841^	0.386	10	42	Ref.	0.004
	0.386	76	84	0.311 (0.139, 0.699)	
IgM anti A2M^824–841^ MDA	0.694	14	38	Ref.	0.105
	0.694	72	88	0.533 (0.249, 1.141)	
IgM anti ApoB100^4022–4040^	0.559	15	38	Ref.	0.157
	0.559	71	88	0.580 (0.272, 1.234)	
IgM anti ApoB100^4022–4040^ MDA	0.581	12	41	Ref.	0.002
	0.581	74	85	0.288 (0.127, 0.652)	
IgM anti A1AT^284–298^	0.345	11	43	Ref.	0.010
	0.345	75	83	0.356 (0.162, 0.785)	
IgM anti A1AT^284–298^ MDA	0.466	8	45	Ref.	<0.001
	0.466	78	81	0.191 (0.077, 0.474)	
IgM anti IGKC^76–99^	0.589	17	36	Ref.	0.790
	0.589	69	90	0.905 (0.434, 1.890)	
IgM anti IGKC^76–99^ MDA	0.252	11	41	Ref.	0.072
	0.252	75	85	0.485 (0.221, 1.067)	

^$^: adjusted by age, sex, and smoke.

**Table 3:** Comparison of AUC, sensitivity, and specificity of autoantibody isotypes against unmodified and malondialdehyde (MDA)-modified peptides in CAD patients compared to HCs with a decision tree classifier (**A**) and random forest classifier (**B**).

**Decision Tree Classifier**			
**IgM anti-ApoB100^4022–4040^ MDA, IgM anti-IGKC^76–99^ MDA, IgM anti-A1AT^284–298^ MDA**			
	Sensitivity (95% C.I.)	Specificity (95% C.I.)	AUC (95% C.I.)
HC v.s. <30%	68.7% (59.3–77.6%)	61.9% (55.4–75.5%)	0.67 (0.55–0.73)
HC v.s. 30–70%	77.4% (66.7–84.5%)	66.4% (58.7–80.9%)	0.76 (0.65–0.82)
HC v.s. >70%	85.7% (73.3–90.1%)	71.7% (68.1–80.6%)	0.81 (0.76–0.86)
**Random Forest Classifier**			
**IgG anti-IGKC^76–99^, IgM anti-IGKC^76–99^ MDA, IgM anti-A1AT^284–298^ MDA**			
	Sensitivity (95% C.I.)	Specificity (95% C.I.)	AUC (95% C.I.)
HC v.s. <30%	74.6% (68.0–79.3%)	64.5% (58.1–72.4%)	0.76 (0.72–0.82)
HC v.s. 30–70%	90.2% (84.5–93.5%)	82.7% (77.9–88.1%)	0.91 (0.87–0.94)
HC v.s. >70%	88.7% (82.7–92.3%)	85.8% (81.0–89.7%)	0.94 (0.88–0.96)

## Data Availability

Not applicable.

## Supplementary Materials

The following are available online at https://www.mdpi.com/article/10.3390/diagnostics11060961/s1, Figure S1: dot plots of plasma concentrations of autoantibody isotypes: immunoglobulin G (IgG) anti-bovine serum albumin (BSA) vs. IgG anti-BSA malondialdehyde (MDA) (A), and IgM anti-BSA vs. IgM anti-BSA MDA (B) in healthy controls (HCs), patients with rheumatoid arthritis (RA) and coronary artery disease (CAD), and patients with RA. Concentrations of antibody isotypes were calculated from calibration curves. Figure S2: dot plots of plasma concentrations of autoantibody isotypes: immunoglobulin G (IgG) anti-alpha-2-macroglobulin (A2M)^824–841^ vs. IgG anti-A2M^824–841^ malondialdehyde (MDA) (A), IgG anti-apolipoprotein B-100 (ApoB100^4022–4040^) vs. IgG anti-ApoB100^4022–4040^ MDA (B), IgG anti-alpha-1-antitrypsin (A1AT^284–298^) vs. IgG anti-A1AT^284–298^ MDA (C), IgG anti-Ig kappa chain C region (IGKC^76–99^) vs. IgG anti-IGKC^76–99^ MDA (D), IgM anti-A2M^824–841^ vs. IgM anti-A2M^824–841^ MDA (E), IgM anti-ApoB100^4022–4040^ vs. IgM anti-ApoB100^4022–4040^ MDA (F), IgM anti-A1AT^284–298^ vs. IgM anti-A1AT^284–298^ MDA (G), and IgM anti-IGKC^76–99^ vs. IgM IGKC^76–99^ MDA (H) in healthy controls (HCs), and coronary artery disease (CAD) patients with <30%, 30%~70%, and >70% stenosis rates using an ELISA. Concentrations of antibody isotypes were calculated from calibration curves. Figure S3: The comparison of ROC curve analysis in each autoantibody. Table S1: the baseline characteristic table for HCs and patients with CAD with <30% stenosis rate versus patients with CAD with 30%~70% and >70% stenosis rate. Table S2: The AUC value of each single antibody with a 95% confidence interval.
