# Supplementary material for: Using Anti-Malondialdehyde Modified Peptide Autoantibodies to Import Machine Learning for Predicting Coronary Artery Stenosis in Taiwanese Patients with Coronary Artery Disease
Source: Diagnostics (Basel). 2021 May 26;11(6):961. doi: 10.3390/diagnostics11060961 (PMC8229983; doi:10.3390/diagnostics11060961)
Supplement: Supplementary file 1 [file diagnostics-11-00961-s001.zip › diagnostics-1209837-supplementary.pdf]

diagnostics

MDPI

Supplementary Materials

Supplementary Table S1. the baseline characteristic table for HCs and patients with CAD with <30% stenosis rate versus patients with CAD with 30%~70% and >70% stenosis rate.

Characteristics	HC and < 30% (*n* = 86)	30%~70% + > 70% (*n* = 126)
Age, years	51.48 ± 16.07	62.67 ± 9.43
Gender, male	55(63%)	93(73%)
Used to smoke	16(18%)	27(21%)
Current smoke	15(17%)	38(30%)
MDA	10.97 ± 4.51	12.71 ± 7.15
MDA adduct	0.21 ± 0.02	0.29 ± 0.82
IgG anti A2M$^{824–841}$	2.02 ± 3.66	1.53 ± 1.6
IgG anti A2M$^{824–841}$ MDA	7.9 ± 12.71	4.67 ± 2.68
IgG anti ApoB100$^{4022–4040}$	3.29 ± 6.87	1.62 ± 1.1
IgG anti ApoB100$^{4022–4040}$ MDA	3.11 ± 9.66	1.67 ± 2.62
IgG anti A1AT$^{284–298}$	3.16 ± 5.61	2.29 ± 1.83
IgG anti A1AT$^{284–298}$ MDA	3.75 ± 3.48	3.61 ± 3.63
IgG anti IGKC$^{76–99}$	2.22 ± 3.03	1.71 ± 3.19
IgG anti IGKC$^{76–99}$ MDA	2.71 ± 4.77	1.4 ± 1.24
IgM anti A2M$^{824–841}$	0.8 ± 0.45	0.59 ± 0.39
IgM anti A2M$^{824–841}$ MDA	1.68 ± 1.09	1.25 ± 0.9
IgM anti ApoB100$^{4022–4040}$	1.22 ± 0.8	1.1 ± 1.13
IgM anti ApoB100$^{4022–4040}$ MDA	1.18 ± 0.81	0.93 ± 0.83
IgM anti A1AT$^{284–298}$	1.14 ± 1.11	1.01 ± 2.19
IgM anti A1AT$^{284–298}$ MDA	1.01 ± 0.53	0.68 ± 0.43
IgM anti IGKC$^{76–99}$	2.24 ± 5.61	5.52 ± 40.03
IgM anti IGKC$^{76–99}$ MDA	0.63 ± 0.53	0.42 ± 0.35

* Mean ± SD for continuous variables, and *n* (%) for categorical variables.

Supplementary Table S2. The AUC value of each single antibody with a 95% confidence interval.

Variables	AUC (95% C.I.)
IgG anti-A2M	0.52 (0.40–0.65)
IgG anti-A2M MDA	0.61 (0.49–0.72)
IgG anti-ApoB100	0.64 (0.52–0.75)
IgG anti-ApoB100 MDA	0.59 (0.48–0.70)
IgG anti-A1AT	0.52 (0.40–0.65)
IgG anti-A1AT MDA	0.58 (0.45–0.71)
IgG anti-IGKC	0.57 (0.45–0.69)
IgG anti-IGKC MDA	0.66 (0.55–0.77)
IgM anti-A2M	0.73 (0.63–0.84)
IgM anti-A2M MDA	0.73 (0.63–0.84)
IgM anti-ApoB100	0.64 (0.53–0.75)
IgM anti-ApoB100 MDA	0.70 (0.59–0.81)
IgM anti-A1AT	0.74 (0.65–0.84)
IgM anti-A1AT MDA	0.83 (0.75–0.91)
IgM anti-IGKC	0.70 (0.59–0.81)
IgM anti-IGKC MDA	0.78 (0.69–0.87)

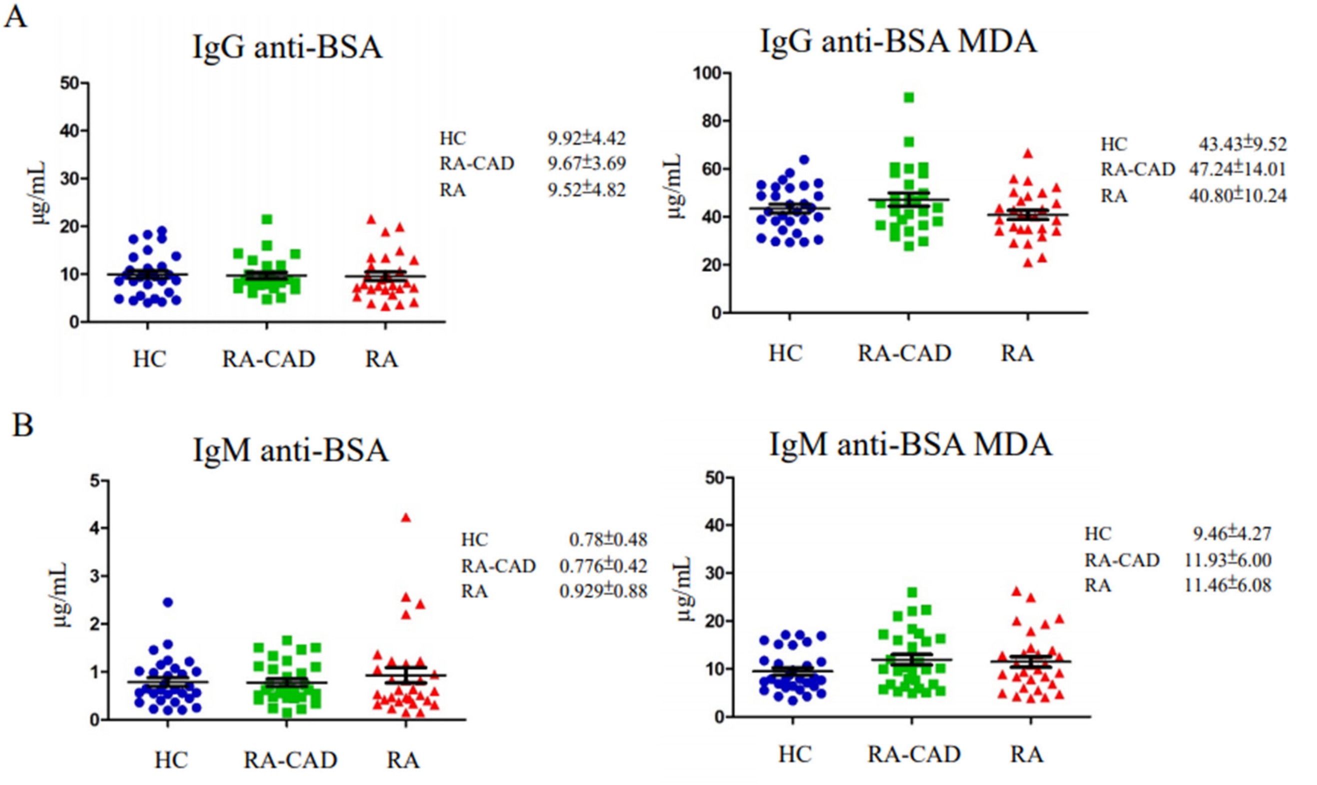

Supplementary Figure S1. Dot plots of plasma concentrations of autoantibody isotypes: immunoglobulin G (IgG) anti-bovine serum albumin (BSA) vs. IgG anti-BSA malondialdehyde (MDA) (**A**), and IgM anti-BSA vs. IgM anti-BSA MDA (**B**) in healthy controls (HCs), patients with rheumatoid arthritis (RA) and coronary artery disease (CAD), and patients with RA. Concentrations of antibody isotypes were calculated from calibration curves.

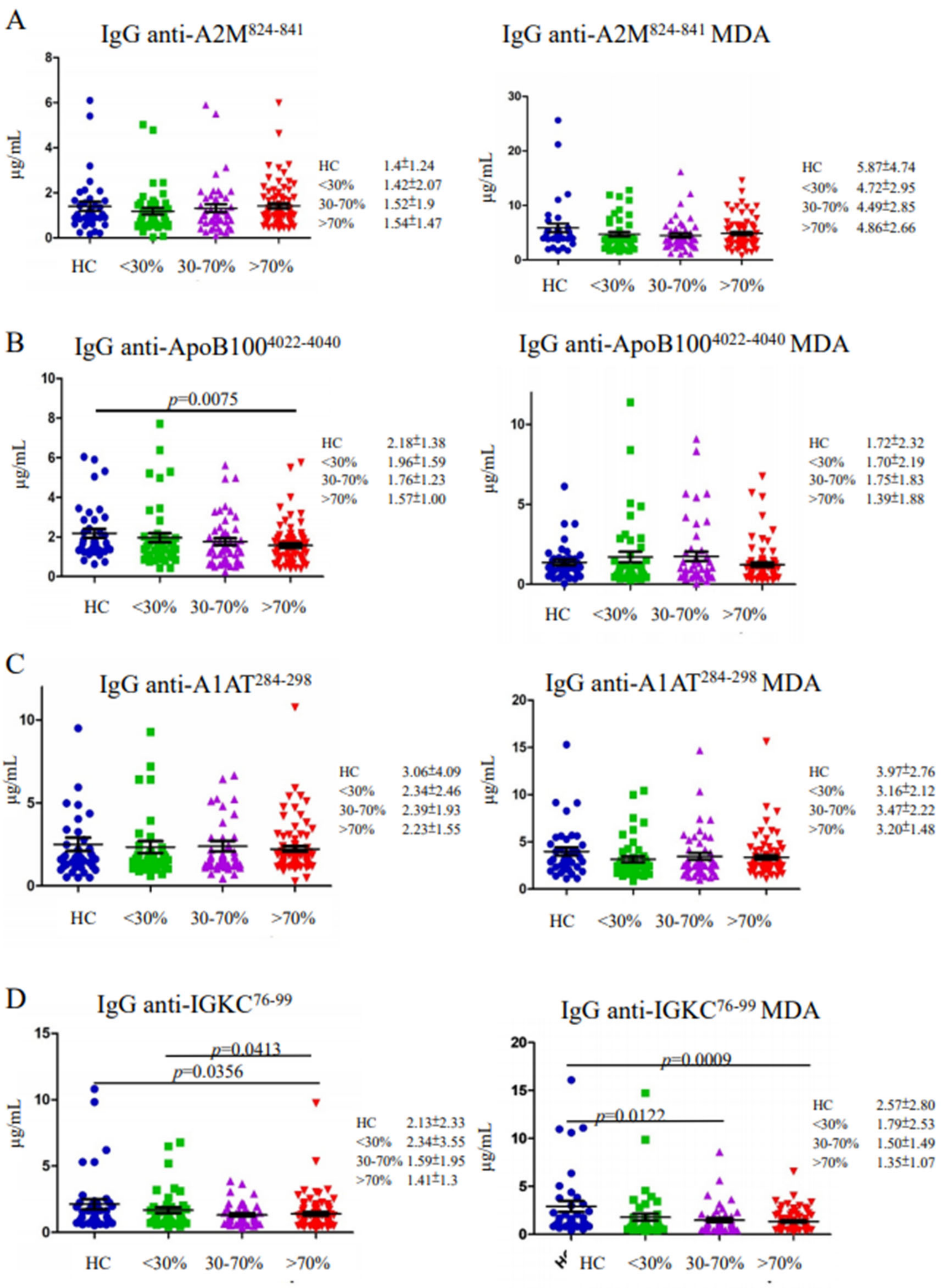
A
IgG anti-A2M824-841
μg/mL
HC <30% 30-70% >70%
HC 1.4±1.24
<30% 1.42±2.07
30-70% 1.52±1.9
>70% 1.54±1.47
IgG anti-A2M824-841 MDA
μg/mL
HC <30% 30-70% >70%
HC 5.87±4.74
<30% 4.72±2.95
30-70% 4.49±2.85
>70% 4.86±2.66
B
IgG anti-ApoB1004022-4040
p=0.0075
μg/mL
HC <30% 30-70% >70%
HC 2.18±1.38
<30% 1.96±1.59
30-70% 1.76±1.23
>70% 1.57±1.00
IgG anti-ApoB1004022-4040 MDA
μg/mL
HC <30% 30-70% >70%
HC 1.72±2.32
<30% 1.70±2.19
30-70% 1.75±1.83
>70% 1.39±1.88
C
IgG anti-A1AT284-298
μg/mL
HC <30% 30-70% >70%
HC 3.06±4.09
<30% 2.34±2.46
30-70% 2.39±1.93
>70% 2.23±1.55
IgG anti-A1AT284-298 MDA
μg/mL
HC <30% 30-70% >70%
HC 3.97±2.76
<30% 3.16±2.12
30-70% 3.47±2.22
>70% 3.20±1.48
D
IgG anti-IGKC76-99
p=0.0413
p=0.0356
μg/mL
HC <30% 30-70% >70%
HC 2.13±2.33
<30% 2.34±3.55
30-70% 1.59±1.95
>70% 1.41±1.3
IgG anti-IGKC76-99 MDA
p=0.0009
p=0.0122
μg/mL
HC <30% 30-70% >70%
HC 2.57±2.80
<30% 1.79±2.53
30-70% 1.50±1.49
>70% 1.35±1.07

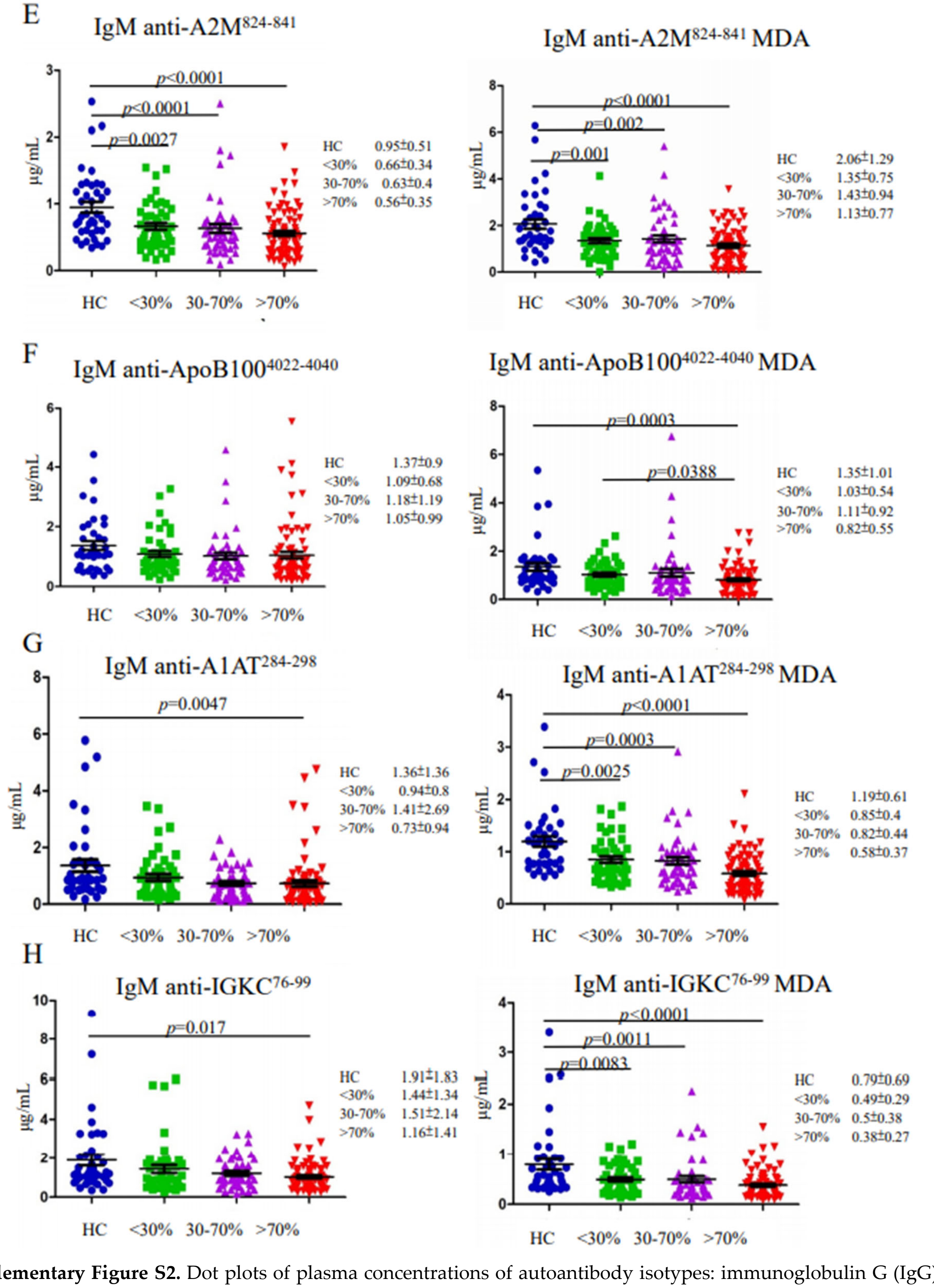

Supplementary Figure S2. Dot plots of plasma concentrations of autoantibody isotypes: immunoglobulin G (IgG) anti-alpha-2-macroglobulin (A2M)$^{824–841}$ vs. IgG anti-A2M$^{824–841}$ malondialdehyde (MDA) (**A**), IgG anti-apolipoprotein B-100 (ApoB100$^{4022–4040}$) vs. IgG anti-ApoB100$^{4022–4040}$ MDA (**B**), IgG anti-alpha-1-antitrypsin (A1AT$^{284–298}$) vs. IgG anti-A1AT$^{284–298}$ MDA (**C**), IgG anti-Ig kappa chain C region (IGKC$^{76–99}$) vs. IgG anti-IGKC$^{76–99}$ MDA (**D**), IgM anti-A2M$^{824–841}$ vs. IgM anti-A2M$^{824–841}$ MDA (**E**), IgM anti-ApoB100$^{4022–4040}$ vs. IgM anti-ApoB100$^{4022–4040}$ MDA (**F**), IgM anti-A1AT$^{284–298}$ vs. IgM anti-A1AT$^{284–298}$ MDA (**G**), and IgM anti-IGKC$^{76–99}$ vs. IgM IGKC$^{76–99}$ MDA (**H**) in healthy controls (HCs), and coronary artery disease (CAD) patients with <30%, 30%~70%, and >70% stenosis rates using an ELISA. Concentrations of antibody isotypes were calculated from calibration curves.

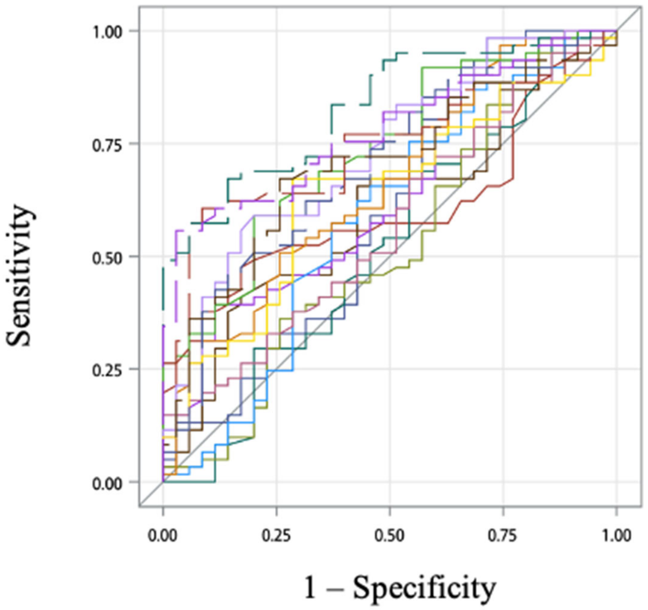

Supplementary Figure S3: The comparison of ROC curve analysis in each autoantibody.

Supplementary Information

1. Supplementary Methods

1.1. Thiobarbituric Acid-Reactive Substance (TBARS) Assay for Malondialdehyde (MDA) Detection

Plasma (10 µL) was mixed with a TBARS solution containing 15% (*w*/*v*) trichloroacetic acid, 0.38% (*w*/*v*) thiobarbituric acid, and 0.25 N hydrochloric acid (HCl). The mixture was heated to 100 °C for 30 min and then cooled on ice for 2 min. After centrifugation, the supernatant was transferred to a plastic plate to measure the absorbance at 535 nm. Interpolation was conducted with MDA to determine the MDA concentration in plasma samples

1.2. Enzyme-Linked Immunosorbent Assay (ELISA) for Quantification of Plasma MDA-Protein Adducts

Diluted plasma samples (10 µg/mL; 100 µL) or MDA-modified bovine serum albumin (BSA; A7906, Sigma) standards (0~10 µg/mL) were added to a flat-bottomed 96-well plate and incubated at 37 °C for 2 h. A 3% BSA solution was used for blocking at room temperature for 1 h after washing the plates with phosphate-buffered saline (PBS) containing 0.05% Tween 20 (PBST). The plates were washed with PBST, and a goat anti-MDA antibody (MBS316059, 1:1000, MyBioSource) was added and incubated at room temperature for 2 h. We washed the plates with PBST, added mouse anti-goat antibody-horseradish peroxidase (HRP; sc2354, 1:10,000, Santa Cruz), and incubated the plates at room temperature for 1 h. After washing the plates, we detected the antibody-HRP with SureBlue ReserveTM TMB Microwell Peroxidase Substrate (Kirkegard & Perry Laboratories, Gaithersburg, MD, USA) and then incubated the plates at room temperature for 30 min. After the color reaction was stopped with 1 N HCl, the absorbance was measured at 450/620 nm. We measured MDA-protein adducts in plasma according to a standard MDA-modified BSA curve. Amounts of MDA-protein adducts are expressed as µg/mL.

1.3. Detection of Autoantibodies against Unmodified and MDA-Modified BSA and Peptides

BSA and four peptides (1 mg/mL) were modified with 0.5 M MDA at 37 °C for 3 h. BSA and peptides (10 μg/mL) were adsorbed in a flat-bottom 96-well plate and incubated at 4 °C overnight. After washing with PBST, 3% BSA was used for blocking. Plates were washed with PBST, and we then added 100-fold diluted plasma and incubated the plates at room temperature for 2 h. After washing the plates, we used rabbit anti-human IgG-HRP (A80-118P, 1:30,000, BETHYL) or rabbit anti-human IgM-HRP (A0420, 1:10,000, Sigma) to detect the autoantibodies against unmodified or MDA-modified peptides at room temperature for 1 h. The antibody-HRP was reacted with the SureBlue ReserveTM TMB Microwell Peroxidase Substrate (Kirkegard & Perry Laboratories, USA) and incubated the plates at room temperature for 15 min. The color reaction was stopped with 1 N HCl, and the absorbance was measured at 450/620 nm. All ELISA experiments were conducted following the ELISA Guidebook [1]. Three sample controls including HCs, and CAD patients with 70% stenosis rates with two replicates were placed in each plate to calculate the percent coefficient of variation (CV%) across wells and plates. An experiment was repeated if the CV% was calculated to be >20%.

References

1. Crowther, J. R. (2000). ELISA Guidebook, The. doi:10.1385/159259049
